# Profiling of the transcriptional response to all-trans retinoic acid in breast cancer cells reveals RARE-independent mechanisms of gene expression

**DOI:** 10.1038/s41598-017-16687-6

**Published:** 2017-11-30

**Authors:** Krysta Mila Coyle, Selena Maxwell, Margaret Lois Thomas, Paola Marcato

**Affiliations:** 1 Department of Pathology, Dalhousie University, Halifax, NS Canada; 2 Department of Microbiology & Immunology, Dalhousie University, Halifax, NS Canada

## Abstract

Retinoids, derivatives of vitamin A, are key physiological molecules with regulatory effects on cell differentiation, proliferation and apoptosis. As a result, they are of interest for cancer therapy. Specifically, models of breast cancer have varied responses to manipulations of retinoid signaling. This study characterizes the transcriptional response of MDA-MB-231 and MDA-MB-468 breast cancer cells to retinaldehyde dehydrogenase 1A3 (ALDH1A3) and all-trans retinoic acid (atRA). We demonstrate limited overlap between ALDH1A3-induced gene expression and atRA-induced gene expression in both cell lines, suggesting that the function of ALDH1A3 in breast cancer progression extends beyond its role as a retinaldehyde dehydrogenase. Our data reveals divergent transcriptional responses to atRA, which are largely independent of genomic retinoic acid response elements (RAREs) and consistent with the opposing responses of MDA-MB-231 and MDA-MB-468 to *in vivo* atRA treatment. We identify transcription factors associated with each gene set. Manipulation of the IRF1 transcription factor demonstrates that it is the level of atRA-inducible and epigenetically regulated transcription factors that determine expression of target genes (e.g. CTSS, cathepsin S). This study provides a paradigm for complex responses of breast cancer models to atRA treatment, and illustrates the need to characterize RARE-independent responses to atRA in a variety of models.

## Introduction

The evolutionarily-conserved retinoid signaling pathway governs expression of hundreds of genes and regulates a wide variety of fundamental biological processes, including differentiation, cell cycle arrest and cell proliferation^1,2^. Retinoid signaling has a controversial role in cancer, with evidence suggesting it can suppress or promote carcinogenesis^1,3–5^, depending on the cancer and the cellular context^4,6–8^. For example, due to their ability to induce differentiation, retinoids are used very successfully to treat acute promyelocytic leukemia^9^ and neuroblastoma^10^. However, attempts to use retinoids and dietary precursors to treat other cancers (including breast cancer) have been unsuccessful and may even promote tumorigenesis^11–13^. A major hypothesis for these clinical disappointments is a failure to consider inter-tumoral heterogeneity^14^. As we better understand the complexities of retinoic acid signaling, we can characterize the divergent responses of breast cancer to retinoids and exploit this heterogeneity for improved cancer therapy.

In breast cancer, the effects of retinoids on cell growth are highly varied and likely depend upon which retinoic acid (RA)-inducible genes are expressed and additional non-genomic effects^3,15–24^. The retinoid signaling pathway is often simplified to production of all-trans retinoic acid (atRA) by aldehyde dehydrogenase 1A (ALDH1A) enzymes, where it translocates to the nucleus and activates nuclear receptors, retinoic acid receptors (RARs) and retinoid X receptors (RXRs). These receptors induce the expression of genes with retinoic acid response elements (RAREs) in their promoters. The human genome contains over 14,000 RAREs; most of which are located in intragenic regions (3,249 RAREs are within 10 kb of genes)^25^. Many initial studies of RA-induced gene expression focused on straightforward induction of genes containing RARE sequences; however, RA-mediated gene expression is significantly more complex and governed by other cellular processes, including the interaction of co-repressors and co-activators. There is strong evidence for hierarchical networks of nuclear receptors facilitating tissue-specific gene expression^26^. Significant choreography is required for the vast transcriptional responses to atRA^27–29^. The high complexity involved in retinoid signaling is unparalleled among nuclear hormone receptor pathways^30^.

Although the majority of evidence supports atRA as a potent anti-cancer therapy, able to suppress proliferation and induce differentiation or apoptosis^31–34^, we and others have demonstrated that RA can also potentiate tumor growth^8,35,36^. Paradoxically, cancer stem cells have high levels of ALDH1A enzymes^37–42^, supporting higher than normal levels of atRA biosynthesis and higher expression of atRA-inducible genes^43^; however, atRA is also used as a differentiating agent which would theoretically eliminate those same cancer stem cells^44–47^. This demonstrates the importance of characterizing cellular responses to atRA in a variety of models, and motivated the current study.

We sought to determine the relationship between the transcriptional profiles associated with ALDH1A3 expression and those corresponding to atRA treatment. We performed mRNA expression arrays (Affymetrix HuGene 2.0ST) with two triple-negative breast cancer (TNBC) cell lines, mesenchymal MDA-MB-231 and basal-like MDA-MB-468 cells. We have previously shown that atRA and ALDH1A3 expression potentiate growth of MDA-MB-231 xenografts, while atRA and ALDH1A3 expression inhibit the growth of MDA-MB-468 xenografts^8^. We identified distinct transcriptional responses with minor overlap to ALDH1A3 and atRA treatment in both cell lines. Among the atRA-inducible genes we identified were a number of known atRA-regulated genes, including keratin 7 (KRT7)^48^, and prostaglandin E synthase (PTGES)^49^. We also identified known regulators of the retinoid signaling pathway, including dehydrogenase reductase 3 (DHRS3)^50^, nuclear receptor interaction protein 1 (NRIP1)^51^, and cytochrome p450 family 26A1 (CYP26A1)^52^.

Since it is established that epigenetic modulation can enhance responses to retinoid-based treatments^53–55^, we provide further evidence that DNA methylation can impact the atRA-inducibility of select genes. On the other hand, the use of the histone deacetylase inhibitor, trichostatin A (TSA), revealed limited contributions of histone acetylation to the regulation of atRA-inducible genes. Very few of the genes we identified as atRA-inducible contained RAREs, and the vast majority of genes containing RAREs were not induced by atRA in either cell line. This again highlights the complexities of differential RA-regulated gene expression, which is cell-type specific and responsible for the diverse cellular effects induced by RA.

Although a major hypothesis for the opposing responses to atRA treatment is differential shuttling of atRA to RARα/β or to peroxisome proliferator-activated receptors (PPAR) β/δ^6,56^, our previous work had indicated that this was not a major contributing factor in the opposing responses to ALDH1A3^8^. This study again indicates that PPARβ/δ-directed transcription is not a major regulator of the pro- or anti-tumor effects of atRA. Instead, we provide evidence for atRA-induced gene expression being predominantly RARE-independent (i.e. cathepsin S, CTSS) and dictated by expression of additional atRA-inducible transcription factors (i.e. interferon regulatory factor 1; IRF1). We demonstrate that IRF1 expression, which is atRA-inducible and epigenetically regulated, is required for full atRA inducibility of CTSS in MDA-MB-231 but not MDA-MB-468. This provides support to a complex network of interactions regulating the context-specific response of breast cancer cells to atRA.

## Results

### ALDH1A3 and atRA activate different transcriptional responses

We have previously characterized that expression of the cancer stem cell marker ALDH1A3 can have opposing effects in two models of TNBC: it can promote the growth of MDA-MB-231 xenografts while limiting the growth of MDA-MB-468 xenografts. We also demonstrated that this was due to differing transcriptional profiles^8^. We determined that the effect of ALDH1A3 on the growth of MDA-MB-231 and MDA-MB-468 xenografts in mice may be attributed to the upstream role of ALDH1A3 in atRA-associated gene expression, as a retinaldehyde dehydrogenase.

**Figure 1:**
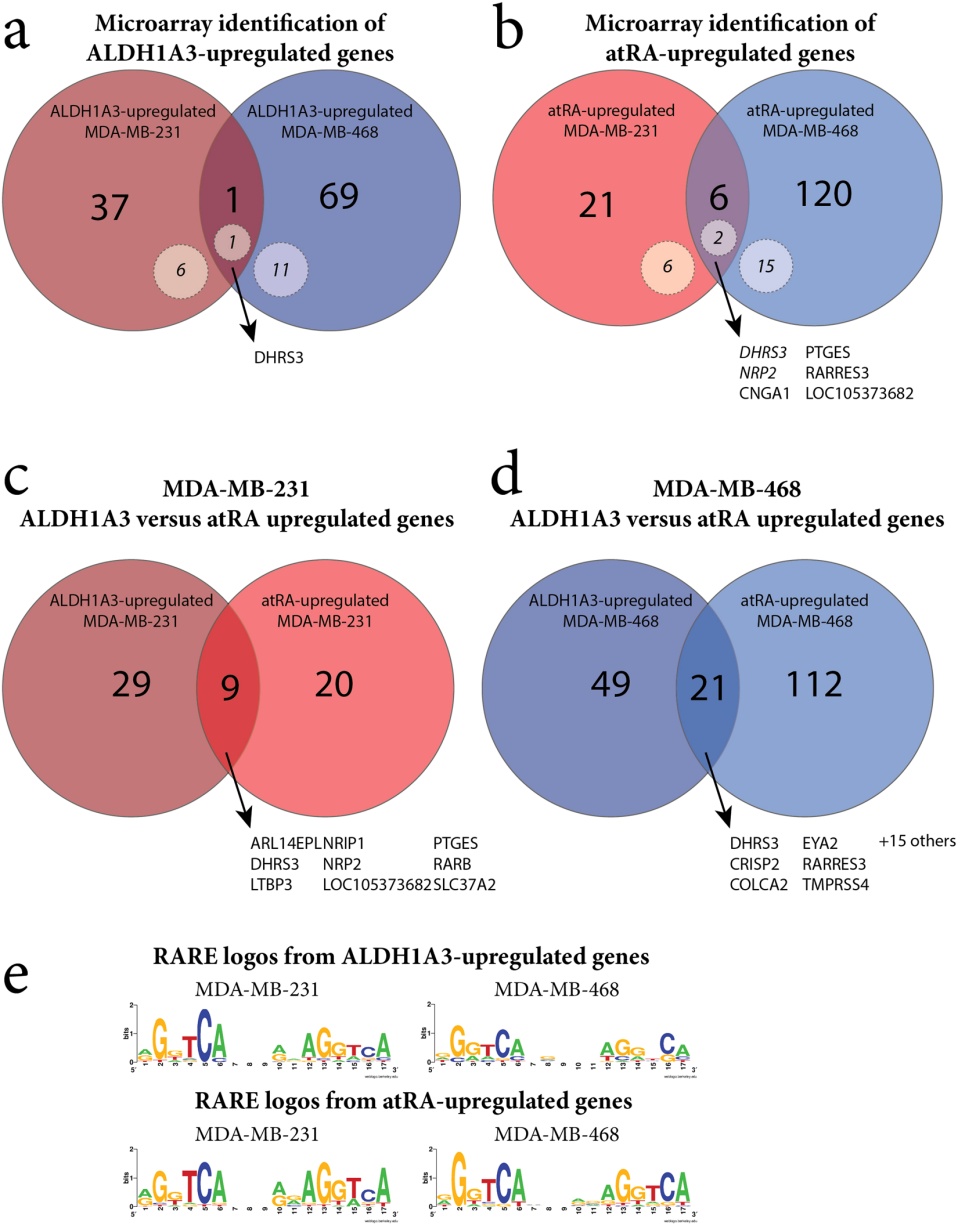
Microarray analysis identifies disparate transcriptional responses to ALDH1A3 manipulation and atRA treatment in MDA-MB-231 and MDA-MB-468 cells. (**a**) Overlap of ALDH1A3-upregulated genes in MDA-MB-231 (ALDH1A3 cDNA/scramble vector) compared to MDA-MB-468 (scramble vector/ALDH1A3 shRNA knockdown). (**b**) Overlap of genes upregulated by 100 nM atRA in MDA-MB-231 and MDA-MB-468. Comparison of ALDH1A3-upregulated and atRA-upregulated genes in (**c**) MDA-MB-231 and (**d**) MDA-MB-468). (**e**) Sequence logos generated from all RAREs identified in associated gene lists.

To assess what proportion of the transcriptional response to ALDH1A3 could be attributed to atRA, we performed gene expression microarrays (Affymetrix HuGene 2.0 ST, GSE103426). We used MDA-MB-231 cells (with or without ALDH1A3 overexpression), MDA-MB-468 cells (with or without ALDH1A3 shRNA), and treated cells with atRA in triplicate. We first identified genes upregulated by ALDH1A3 (knockdown or overexpression) from our microarray data in both MDA-MB-231 and MDA-MB-468 (Fig. 1a, Supplemental File 1). The overlap between the two cell lines is small (DHRS3), suggesting that ALDH1A3 can activate divergent gene expression profiles in these two selected cell lines. We selected a number of these genes for validation (Supplementary Figure S1). Of note, qPCR validation identified additional genes which were regulated by ALDH1A3 in both cell lines (i.e. RARB, SCEL, PTGES, as in Supplementary Figure S1).

We similarly identified genes upregulated by atRA treatment in MDA-MB-231 and MDA-MB-468 (Fig. 1b, Supplemental File 1) and validated the changes in expression of a number of these genes (Supplementary Figure S1). In almost all cases, qPCR validation supported the trends observed by microarray. Of note, according to the microarray data, SRPX2 was only upregulated by atRA in MDA-MB-468 (and not MDA-MB-231); however, in qPCR validation, even with atRA treatment, SRPX2 was below the limit of detection in MDA-MB-468 and was significantly induced by atRA in MDA-MB-231 (Supplementary Figure S1). The small number of genes consistently upregulated by atRA treatment in these two cell lines (Fig. 1b) demonstrates that atRA can also activate divergent gene expression profiles depending on the cellular context (i.e. MDA-MB-231 versus MDA-MB-468).

To test our hypothesis that a substantial proportion of genes upregulated by ALDH1A3 could be attributed to transcriptional activation by atRA, we compared the ALDH1A3-upregulated genes with the atRA-upregulated genes (Fig. 1c,d). The overlap between the ALDH1A3-regulated genes and the RA-regulated genes is small but relevant in both MDA-MB-231 (Fig. 1c) and MDA-MB-468 (Fig. 1d). This demonstrates that exogenous application of atRA is not equivalent to manipulation of ALDH1A3 expression, and suggests that ALDH1A3 may have atRA-independent effects on gene transcription.

It is possible that the differences between atRA-induced gene expression and ALDH1A3-induced gene expression is due to differences in the ‘dosing’ of atRA between exogenous application of atRA and expression of ALDH1A3 (i.e. genes may be upregulated by ALDH1A3 but not exceed the threshold set for differential expression). To eliminate this possibility, we compared all genes which were upregulated by either atRA or ALDH1A3 in either MDA-MB-231 or MDA-MB-468 (Supplementary Figure 2). While there are a number of genes which are upregulated by both ALDH1A3 and atRA to different extents, this does not explain the majority of differences in gene expression. This indicates that a relevant subset of the ALDH1A3-regulated transcriptional response can be attributed to atRA, but that there is a significant proportion which cannot be attributed to atRA.

### Transcriptional regulation by ALDH1A3 and atRA is largely RARE-independent

We had previously described that the transcriptional response to ALDH1A3 was largely RARE-independent^8^. To confirm these findings in a new data set, we again identified genes with RAREs among those upregulated by ALDH1A3 in either MDA-MB-231 or MDA-MB-468 cells (dotted circles in Fig. 1a). We compared the RARE sequences within 10 kb of a RARE DR5 predicted by in silico findings from Lalevée *et al*.^25^ and oPOSSUM^57^ using sequence logos (Fig. 1e, Supplementary Table S2). The small number of genes identified within 10 kb of a RARE DR5 (7 or 18.4% in MDA-MB-231, 12 or 17.1% in MDA-MB-468) and the nearly identical sequence logos allow us to conclude that the ALDH1A3-regulated transcriptional response is largely RARE-independent.

Given that ALDH1A3 appears to regulate the expression of a subset of genes independent of atRA, we hypothesized that a greater proportion of the transcriptional response to atRA would be RARE-dependent. Among the atRA-upregulated genes, we again identified those genes which were located within 10kb of a RARE DR5 using data from Lalevée *et al*.^25^ and oPOSSUM^57^ (Supplementary Table S2). Only a small number of genes (8 or 29.6% in MDA-MB-231 and 17 or 13.5% in MDA-MB-468) were called as within 10 kb of a RARE DR5. The small percentage of genes within regulatory distance of a RARE suggests that atRA-induced gene expression in these TNBC cell lines is primarily (>70%) RARE-independent. We generated TF logos from these genes (Fig. 1e). The logos all demonstrate a high degree of similarity with the core hexameric motif which comprises the RARE direct repeat, separated by 5 nucleotides (DR5): (A/G)G(G/T)TCA^58^. This is expected due to the methods to identify RARE-containing genes. Additionally, we noted no substantial variation between cell lines or between ALDH1A3-regulated or atRA-regulated genes. This suggests that there are no substantial preferences for minor variations in nucleotide sequence within the RARE DR5.

### Epigenetic silencing restricts transcriptional response to atRA

The utility of nuclear receptors depends on their ability to bind target DNA and activate transcription. We previously found that DNA methylation could restrict the expression of a number of ALDH1A3 -inducible genes^8^. Therefore, we hypothesized that DNA methylation may restrict the expression of potential atRA-inducible genes by preventing the induction of transcription and resulting in the disparate gene expression induced by atRA in MDA-MB-468 and MDA-MB-231 cells. To address this possibility, our microarray experiment also included three biological replicates treated with the cytidine analog and DNA methyl-transferase inhibitor, 5-aza-2′-deoxycytidine (also known as decitabine, DAC), alone or in combination with atRA treatment.

**Figure 2:**
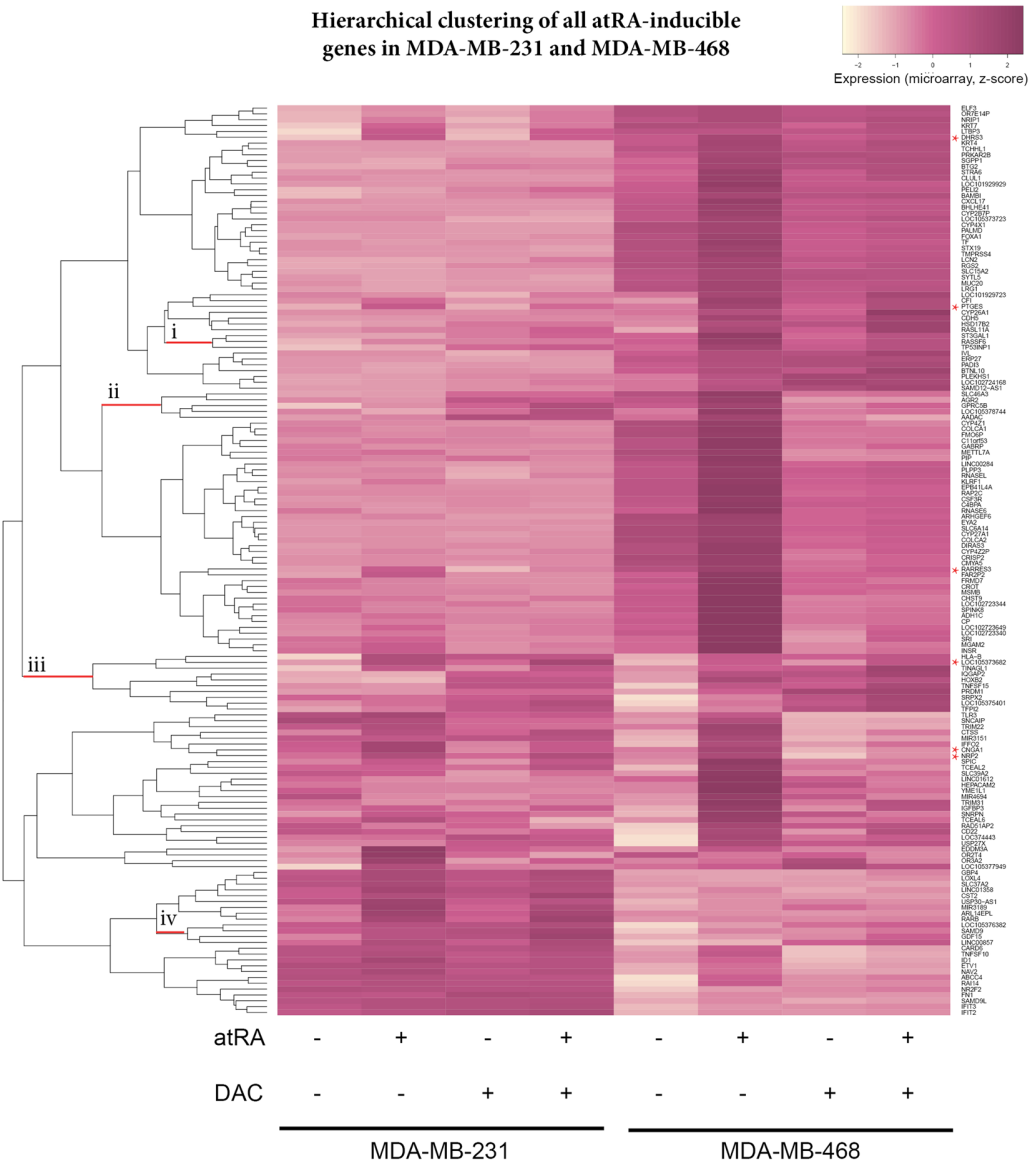
DAC treatment does not align atRA-induced transcriptional profiles. Hierarchical clustering (heatmap.2, gplots) of microarray expression values from MDA-MB-231 and MDA-MB-468 cells treated with atRA, DAC, or both demonstrate that the use of DAC did not align the RA-inducible transcriptional profiles in these cell lines. Genes which were commonly upregulated in both cell lines are indicated by * on the right-hand side, while limited clusters of genes which displayed DAC-permissive atRA inducibility are indicated by lowercase Roman numerals. These genes are described in more detail in Supplementary Table S3.

**Figure 3:**
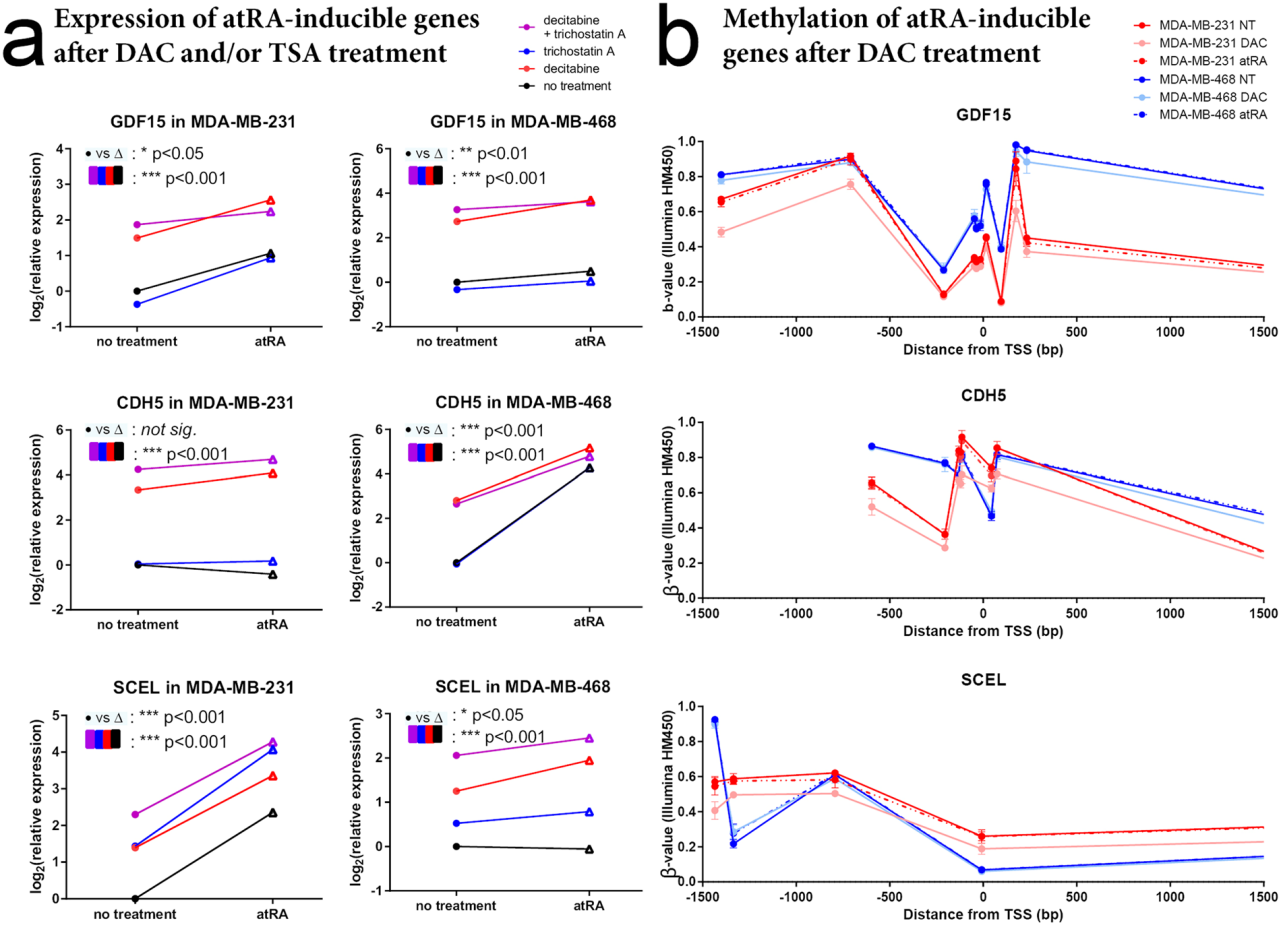
Decitabine does not restore atRA inducibility of specific genes between cell lines. (**a**) MDA-MB-231 and MDA-MB-468 cells were treated with atRA, DAC, and/or TSA and relative expression of GDF15, CDH5, and SCEL were determined by qPCR. A two-way analysis of variance was used to compare the effect of atRA treatment to the effects of DAC and/or TSA treatment (n = 4, *p < 0.05, **p < 0.01, ***p < 0.001). (**b**) β-values representing the relative methylation (Illumina HM450 arrays) of distinct CpG sites in MDA-MB-231 and MDA-MB-468 cells treated with DAC are compared within 1500 bp of the transcription start site (TSS) (n = 3, GSE103425).

Next, we clustered the atRA-inducible genes in either cell lines based on their expression following atRA, DAC, or combination treatment (Fig. 2). Of note, the 6 commonly upregulated genes did not cluster together (indicated with a red star). Although globally, DAC did not enable atRA induction, we identified several clusters of genes where treatment with DAC appeared to enable atRA induction (indicated as “i”, “ii”, “iii”, and “iv”, Fig. 2). The genes in these clusters, and the presence or absence of a RARE, are listed in Supplementary Table S3. We selected several genes from these clusters for further investigation following treatment with atRA, DAC, and TSA, which inhibits class I and II histone deacetylases (HDACs). Among the genes we selected, GDF15 becomes atRA-inducible in the presence of DAC in MDA-MB-468 cells; CDH5 becomes atRA-inducible in the presence of DAC in MDA-MB-231 cells; and SCEL becomes atRA-inducible in the presence of DAC in MDA-MB-468 cells (Fig. 3a). The remainder of the genes investigated show no significant contributions of DAC treatment to the effects of atRA (e.g. GPRC5B and IQGAP2, Supplementary Figure S3); however, there is a clear role for epigenetic regulation of expression in all genes examined.

To further query the methylation of these genes, we utilized the HM450 array for MDA-MB-231 and MDA-MB-468 cells, with or without DAC treatment (GSE103425). We investigated the DNA methylation of GDF15, CDH5, and SCEL, using probes located +/−1500 bp from the transcription start site (TSS) (Fig. 3b). Notably, while DAC has a substantial effect on expression of GDF15 in MDA-MB-468, only minor changes in methylation of GDF15 are seen with DAC treatment. The opposite is seen in CD22 and HOXB2, where DAC has substantial effects on DNA methylation (Supplementary Figure S4), with no corresponding changes in gene expression (Supplementary Figure S4). Among those methylation sensitive genes (i.e. GDF15, CDH5, and SCEL), we report no effect of atRA treatment on DNA methylation as measured by the HM450 array (Fig. 3b). TSA did not appear to play a significant role in gene expression (except for TINAGL1 and PRDM1, Supplementary Figure S4) which suggests that histone acetylation by HDACs 1, 3, 4, 6, or 10 is not a major contributory factor to the divergent gene expression profiles. Since these data suggested that DAC was unable to fully align the divergent transcriptional profiles, and that neither DNA methylation nor histone acetylation of the genes were concordant with the differences in mRNA expression, we then hypothesized that the expression of additional regulatory factors could be responsible for the differential transcriptional responses to atRA seen in these two TNBC models.

### IRF1 is associated with atRA-upregulated genes in MDA-MB-231 and MDA-MB-468 cells

**Figure 4:**
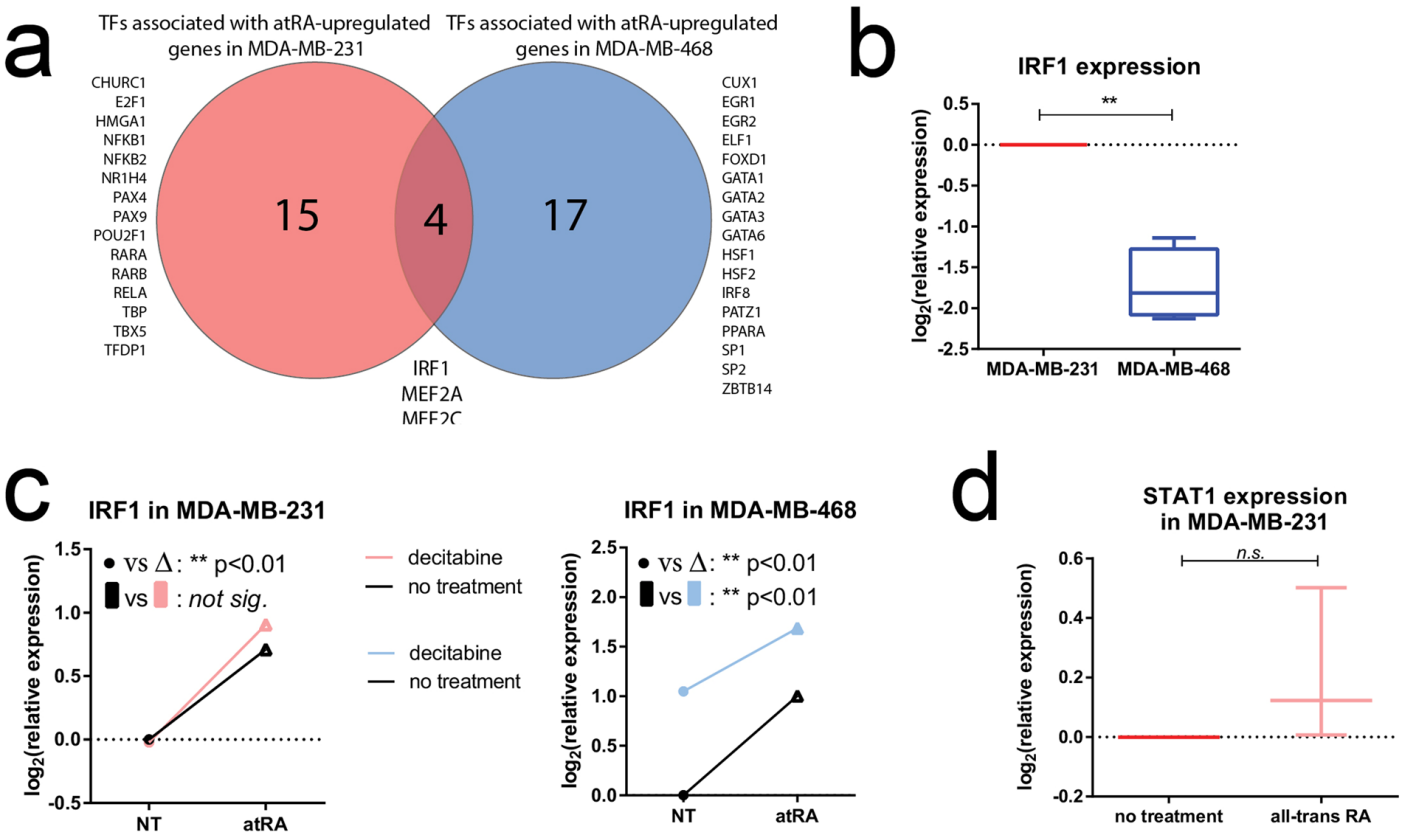
IRF1 is an atRA-inducible transcription factor. (**a)** PASTAA analysis of transcription factor affinities identified disparate transcription factors associated with atRA-inducible genes in MDA-MB-231 as compared to MDA-MB-468. (**b**) qPCR was used to detect expression of IRF1 in MDA-MB-231 and MDA-MB-468 cells. (**c**) Relative expression of IRF1 following atRA and DAC treatment in MDA-MB-231 and MDA-MB-468 cells was determined by qPCR. A two-way analysis of variance was used to compare the effect of atRA treatment to the effect of DAC treatment (*p < 0.05, **p < −0.01). (**d**) The expression of STAT1 was measured in MDA-MB-231 following atRA treatment and compared using a paired student’s t-test.

We used a discovery-motivated approach to identify potential regulatory transcriptional factors associated with the genes upregulated by atRA treatment in either MDA-MB-231 or MDA-MB-468. We used PASTAA (Predicting ASsociated Transcription factors from Annotated Affinities)^59^ to identify transcription factors with high binding affinities within our upregulated gene lists. The top matrices in each cell line were identified and the corresponding transcriptional signatures. Using a cut-off of association >2.0 and p < 0.05, 27 matrices were identified in MDA-MB-231 and 30 matrices were prioritized in MDA-MB-468 cells. This set of matrices corresponded to two lists of 19 and 21 transcription factors in MDA-MB-231 and MDA-MB-468 cells, respectively (Fig. 4a). Given that the genes upregulated by atRA were quite distinct (Fig. 1b), it was not surprising that the transcription factors identified with high affinities for the gene lists from each cell line were also distinct. We also examined the transcription factors associated with down-regulated genes (summarized in Supplementary Figure S5). Those with high association scores are consistent with previously published data which suggest that atRA can downregulate genes by interfering with promiscuous transcription factors such as AP1 (composed of a FOS/JUN heterodimer)^60,61^.

Of note, the retinoic acid receptors α and β (RARα and RARβ) were only identified with high affinity in the MDA-MB-231 gene list. We did not investigate this canonical pathway as there is a substantial body of evidence indicating that methylation of RARβ contributes to differences in atRA-inducible transcription^62–64^. It is also of interest that PPARα was only identified above our threshold in the MDA-MB-468 gene list. This suggests that redirection of atRA through FABP5 to PPARβ/δ is not a predominant mechanism for the promotion of tumor growth and proliferation by atRA in our model.

We identified interferon regulatory factor 1 (IRF1) and the myocyte enhancer factor 2 (MEF2) family of transcription factors as significantly associated with the gene lists from both MDA-MB-231 and MDA-MB-468 cells. atRA is known to activate MEF2C^65^ and to induce expression of IRF1^66^. To prioritize further experiments, we then examined the expression of all indicated transcription factors in the microarray data (Supplementary Figure S6). We selected IRF1 for further characterization due to its differential expression between MDA-MB-231 and MDA-MB-468 cells, an increase in expression following DAC treatment in MDA-MB-468 cells, and its RA-inducibility in both cell lines (Supplementary Figure S6). In contrast, the MEF2 family of transcription factors showed no substantive response to RA (Supplementary Figure S6).

IRF1 has been previously characterized with a RARE DR5; however, induction of IRF1 by atRA appears to be mediated by an interferon regulatory element^67^. When we validated the expression by qPCR, we confirmed that IRF1 was more highly expressed in MDA-MB-231 cells (Fig. 4b), and could be significantly induced by atRA in both cell lines (Fig. 4c). Increased expression with DAC was only seen in MDA-MB-468 cells (Fig. 4c). This suggests that differential expression of IRF1 may be a contributing factor to the divergent gene expression profiles in two TNBC cell line models. To confirm previous reports that the induction of IRF1 by atRA was independent of STAT1 (signal transducing activator of transcription 1)^68^, we measured STAT1 mRNA expression in MDA-MB-231 cells following atRA treatment (Fig. 4d). We observed no effect of atRA on STAT1 mRNA, suggesting that if atRA affects IRF1 expression via STAT1, it does so non-genomically.

### IRF1 expression is necessary for atRA induction of CTSS expression

**Figure 5:**
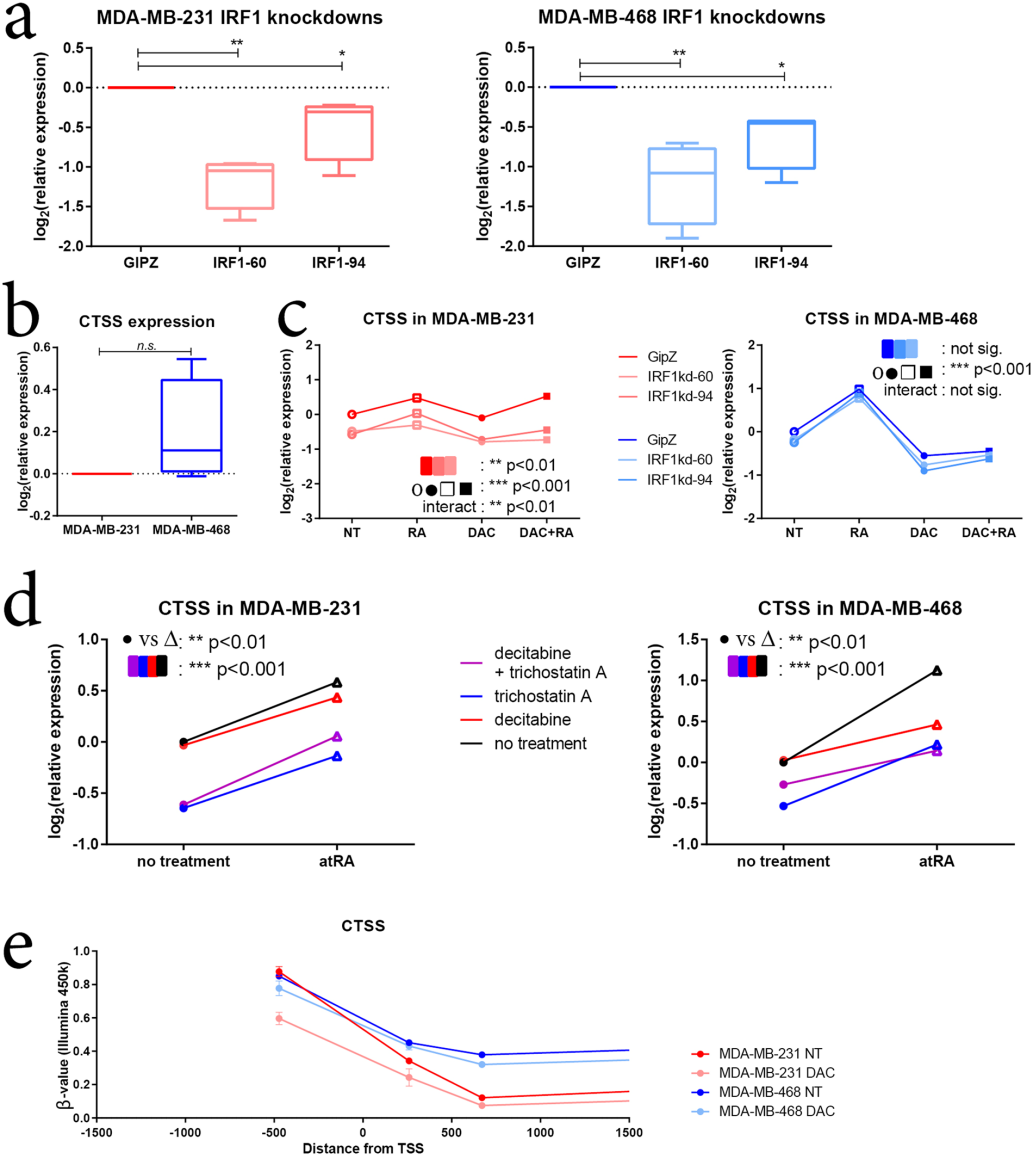
IRF1 expression is required for CTSS expression in MDA-MB-231 cells. (**a**) shRNA knockdowns of IRF1 were generated in MDA-MB-231 and MDA-MB-468. Values were compared using a one-way analysis of variance with repeated measures (n = 4). (**b**) CTSS expression was measured by qPCR and compared between MDA-MB-231 and MDA-MB-468 cells using a paired student’s t-test (n = 4). (**c**) CTSS expression was measured in shRNA knockdowns following treatment with atRA and/or DAC. A two-way analysis of variance was used to determine the effect of IRF1 knockdown compared to atRA/DAC treatment (n = 4). (**d**) MDA-MB-231 and MDA-MB-468 cells were treated with DAC and/or TSA and CTSS expression was measured by qPCR. Values were compared with a two-way analysis of variance (n = 4). (**e**) β-values representing the relative methylation (Illumina HM450 arrays) of distinct CpG sites in MDA-MB-231 and MDA-MB-468 cells treated with DAC are compared within 1500 bp of the transcription start site (TSS) (n = 3, GSE103425). For all statistical comparisons, *p < 0.05, **p < 0.01, ***p < 0.001.

To further validate the role of IRF1 in differential RA transcriptional responses, we generated two shRNA knockdowns of IRF1 in MDA-MB-231 and MDA-MB-468 cells with varying efficiencies (Fig. 5a). We then treated these vector-bearing cell lines with atRA and/or DAC, and measured the expression of several known IRF1 target genes (retinoic acid receptor response protein 3, RARRES3; guanylate binding protein 4, GBP4; and cathepsin S, CTSS)^69,70^. Of these genes, RARRES3 and GBP4 possess RARE DR5s^25,71^. We demonstrate that although RARRES3 is more highly expressed in MDA-MB-468 cells, IRF1 knockdown has similar effects on its expression in both MDA-MB-231 and MDA-MB-468 cells (Supplementary Figure S7). Next, although GBP4 is more highly expressed in MDA-MB-231, IRF1 knockdown has similar effects on its expression in both cell lines (Supplementary Figure S7). We also examined the expression of TNFSF10 (tumor necrosis factor super family member 10; TNF-related apoptosis-inducing ligand, TRAIL) an important target of IRF1 transcription factor activity^72^, and determined that its expression in both MDA-MB-231 and MDA-MB-468 cells is largely IRF1-independent (Supplementary Figure S7).

**Figure 6:**
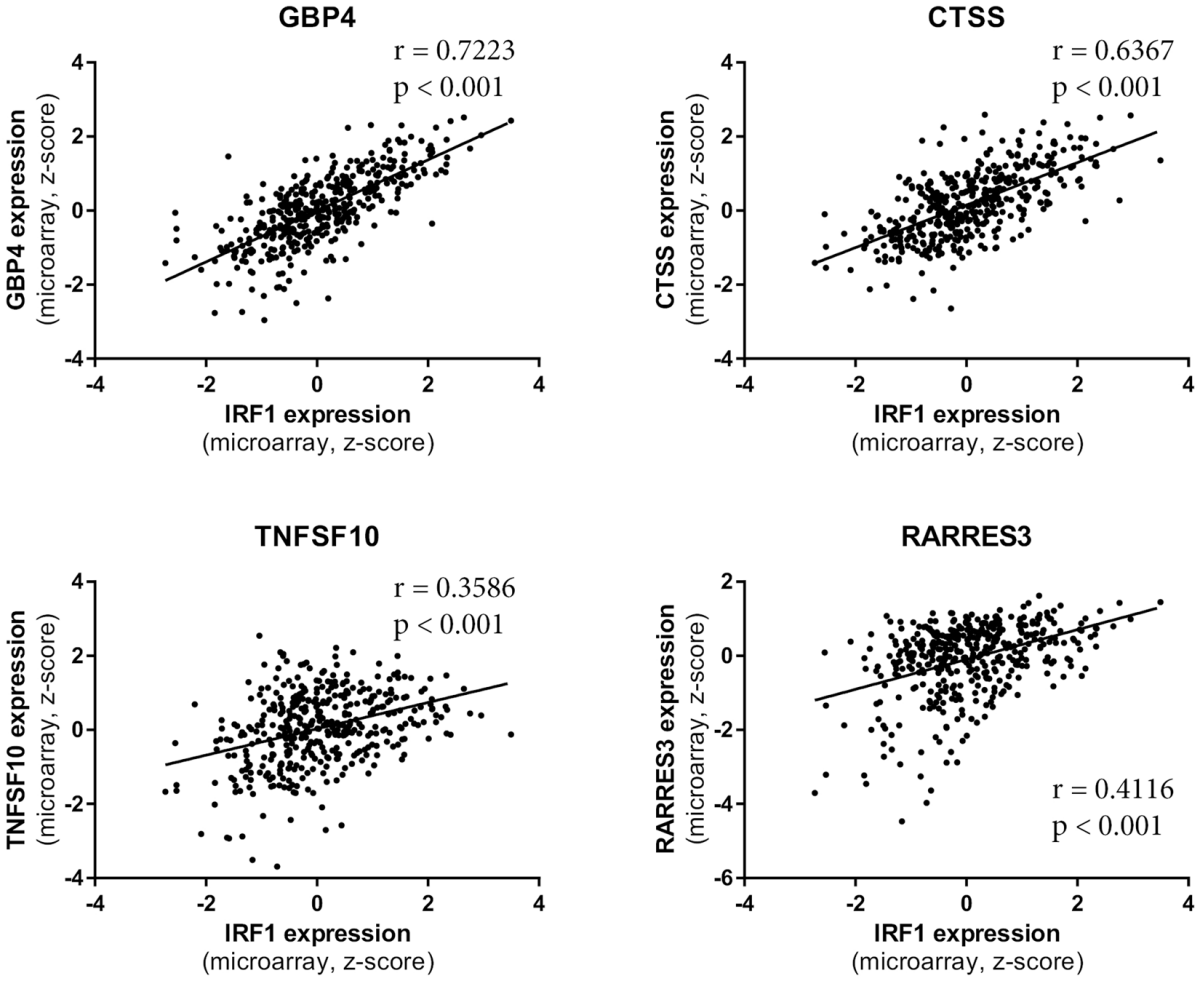
IRF1 expression strongly correlates with CTSS and GBP4 expression in breast cancer patient tumors. The expression of IRF1 target genes CTSS, GBP4, TNFSF10, and RARRES3 in 421 breast cancer patient tumors^90^ are plotted against IRF1 expression. Correlation and significance are indicated for each plot.

Of note, IRF1 expression was required for CTSS expression in MDA-MB-231 but not in MDA-MB-468 cells (Fig. 5b). IRF1 expression was also required for full atRA-induced CTSS expression in MDA-MB-231 cells. While CTSS displays differential methylation between MDA-MB-231 and MDA-MB-468 (Fig. 5c), neither DAC nor TSA induced changes in expression (Fig. 5d). Our findings that IRF1 expression contributes to the regulation of RARRES3, GBP4, and CTSS, but not to the regulation of TNFSF10, are further validated by mRNA expression data from breast cancer patients. We observe strong correlations of IRF1 expression with GBP4 and CTSS, but only a weak correlation with RARRES3 and TNFSF10 expression (Fig. 6).

In addition to the contributions of DNA methylation to the expression of atRA-inducible transcripts such as GDF15, CDH5, and SCEL, these findings demonstrate that baseline expression of additional regulatory transcriptional factors such as IRF1 can be sufficient to activate divergent transcriptional profiles in cells.

## Discussion

Mechanisms of RARE-independent RA-mediated gene regulation have been proposed. For example, atRA can regulate gene expression via *trans*-mechanisms, such as interaction of the RARs with the transcription factors of other signaling pathways (e.g. estrogen receptor, ER, in ER^+^ breast cancer cells)^73,74^. RA activated RARs can also act as epigenetic modifiers, altering chromatin structure and thus gene expression^75–77^. Through interactions with transcription complex proteins, RA bound RARs influence the addition or removal of epigenetic marks, such as histone methylation and acetylation, and DNA methylation. Interestingly, we did not see evidence of changes in gene methylation upon induction of gene expression by atRA, while we did note instances where DNA methylation contributes to differential RA-mediated gene expression. However, while promoter methylation contributes to the restricted atRA-inducible transcriptional responses in TNBC MDA-MB-231 and MDA-MB-468 cells, our data suggests that it is the expression of other regulatory factors such as IRF1 which mediate the expression of genes without RAREs. In fact, both atRA and DAC appear to modulate IRF1 expression in these cell lines. While the majority of this work focused on coding transcripts, several long non-coding RNAs were identified including linc00857 (upregulated in MDA-MB-231). Further work on understanding the regulation of non-coding transcripts by RA, and their subsequent regulation of the coding genome, will be important to contextualize the full cellular genomic context and cellular response to RA. This provides further evidence that the response of breast cancer cells to atRA is primarily non-canonical, and indicates an area needing additional investigation if the full potential of atRA as a cancer therapeutic is to be achieved.

We further characterized the transcriptional response to ALDH1A3 manipulation by comparing it to atRA and DAC treatment in two TNBC cell lines. Although ALDH1A3 is characterized primarily as a retinaldehyde dehydrogenase, we identified limited overlap between the ALDH1A3-upregulated and the atRA-upregulated transcriptional profiles in both cell lines. While we and others have demonstrated a clear role for ALDH1A3 in initiating retinoid signaling and affecting the expression of RARE-containing genes including RARβ, the tumor suppressor gene RARRES1, and tissue transglutaminase^8,42,78^, the sole contribution of ALDH1A3 as a retinaldehyde dehydrogenase in breast cancer and the stem cell phenotype (cancerous or non-cancerous) may be overstated^8,41,44,79–81^. Alternate functions of ALDH1A3 may contribute to breast cancer progression and stem-cell activity. We note that atRA has been used as an ALDH inhibitor in cancer stem cell studies^45,82^; however, considering that we demonstrate some overlap between ALDH1A3 and atRA regulated genes in breast cancer cells, the regulation of ALDH enzymes with atRA is likely to confound studies of the biological effects of ALDH1A3. The regulation of ALDH1A3 by atRA is likely to be context-dependent^83^.

One possible explanation is that other potential substrates of ALDH1A3 may further account for the observed discrepancies between transcriptional profiles in this study, including those involved in glycolysis^84^ and the oxidation of lipid peroxidation-derived aldehydes^85,86^. Additionally, the mouse ALDH1A3 (also known as RALDH3) has a high affinity for octanal, decanal, and hexanal^87^, which can affect gene expression^88^. There is a high degree of structural similarity between the ALDH1A isoforms, so ALDH1A3 may have yet uncharacterized enzymatic substrates, including 9-cis retinal^43^. These additional substrates may explain the lack of concordance between the ALDH1A3-induced and RA-induced transcriptional profiles.

We demonstrate that the pleotropic effects of atRA on breast cancer cells are related to non-genomic and multi-layered pathways; and restricting analysis of atRA-responsiveness in breast cancer to canonical RARE-containing genes limits the biological relevance. The cellular context of breast cancer cells (i.e. the expression of atRA-inducible transcription factors) is a major contributing factor to the divergent responses observed to atRA.

## Methods

### Cell lines, vectors, and reagents

MDA-MB-231 and MDA-MB-468 cells were obtained from the American Type Culture Collection (ATCC) and cultured in Dulbecco’s Modified Eagle’s Medium (DMEM) with 10% fetal bovine serum and 1x antibiotic-antimycotic (Invitrogen). DDC Medical authenticated the cell lines by short tandem repeat (STR) profiling at 17 loci and verified them to be mycoplasma-negative (last performed 2015).

All-trans retinoic acid (atRA, Sigma) was used at 100 nM for 18 h. 5-aza-2′-deoxycitidine (decitabine, DAC, Sigma), was used at 1 μM for 72 h and replaced every 24 h. Trichostatin A (TSA, Sigma) was used at 100 nM for 18 h. When used in combination, atRA and TSA were added for the last 18 h of treatment.

IRF1 shRNA knockdown clones were generated using the pGipZ lentiviral vector packaged in HEK293T cells following standard protocols (IRF1kd-60: V3LHS_412360; IRF1kd-94: V2LHS_133394; Dharmacon). ALDH1A3-overexpressing and ALDH1A3 knockdown clones were generated and validated as previously described^8,41^.

### Quantitative PCR

Total RNA was extracted using Trizol reagent and the PureLink RNA kit (Invitrogen) with DNase treatment. Equal amounts of RNA were reverse transcribed using iScript (BioRad) and quantitative real-time PCR (qPCR) was performed using gene-specific primers (Supplementary Table S1). Standard curves for each primer set were generated, and primer efficiencies were incorporated into the CFX Manager software (Bio-Rad). mRNA expression of all samples was calculated relative to two reference genes [glyceraldehyde 3-phosphate dehydrogenase (GAPDH) and β-2-microglobulin (B2M)], and an indicated control sample. Relative mRNA expression was log-2 transformed prior to plotting and statistical analysis.

### Gene expression profiling

MDA-MB-231 MSCV and ALDH1A3-overexpression cells, and MDA-MB-468 SMP and ALDH1A3-shRNA cells were treated with atRA and/or DAC as described in triplicate. Sample preparation, amplification, hybridization to the Affymetrix HuGene 2.0 ST array, and data collection were performed by The Centre for Applied Genomics at the Hospital for Sick Children (Toronto, Ontario, Canada) and can be accessed by Geo Accession Reference GSE103426. Data was analyzed in the R environment using the oligo package with RMA normalization. Genes which were up-or down-regulated more than 1.6-fold (log_2_ = 0.678) at a significance level of p < 0.01 were considered differentially expressed.

### Methylation profiling

DNA was collected from untreated and DAC-treated MDA-MB-231 and MDA-MB-468 cells using the PureLink DNA kit (Invitrogen). Methylation analyses using the HM450 bead chip array (Illumina) was performed by The Centre for Applied Genomics including bisulfite conversion, hybridization, background subtraction, and normalization (Geo Accession GSE103425). β-values for Illumina probes near each gene of interest were extracted from the data, and location determined relative to the transcription start site (TSS).

### Transcription factor logos

Genes with retinoic acid response elements (RAREs) were identified from published data (Lalevee *et al*.^25^) or from the oPossum database^57^ within 10 kb of the TSS of the gene of interest. RARE sequences were entered into WebLogo^89^ with default settings. Where a gene was identified with more than 1 RARE within 10 kb of the TSS, all identified RARE sequences were utilized for logo generation.

### Statistical analyses

All statistical analyses were calculated in GraphPad Prism 6. Paired t-tests were used to compare single treatments, one-way ANOVA was used to compare multiple vectors, and two-way ANOVA was used to compare combinations of treatments and/or vectors. Pearson’s correlations were calculated for TCGA data appearing in Fig. 6. For all comparisons, *p < 0.05, **p < 0.01, ***p < 0.001.

### Data availability

The microarray and DNA methylation datasets generated during and analysed during the current study are available in the Geo repository as series GSE103427. Data from The Cancer Genome Atlas (TCGA)^90^ were analyzed and extracted from the cBioportal interface^91,92^. Other data and samples are available from the corresponding author on reasonable request.

## Electronic supplementary material

Supplementary Tables and Captions

File 1
