# Supplementary material for: Profiling of the transcriptional response to all-trans retinoic acid in breast cancer cells reveals RARE-independent mechanisms of gene expression
Source: Sci Rep. 2017 Nov 30;7:16684. doi: 10.1038/s41598-017-16687-6 (PMC5709375; doi:10.1038/s41598-017-16687-6)
Supplement: Supplementary file 1 — Supplementary Tables and Captions [file 41598_2017_16687_MOESM1_ESM.pdf]

Profiling of the transcriptional response to all-trans retinoic acid in breast cancer cells reveals RARE-independent mechanisms of gene expression

Krysta Mila Coyle[1], Selena Maxwell[2], Margaret Lois Thomas[1], Paola Marcato[1,2]

[1] Department of Pathology, Dalhousie University, Halifax NS Canada

[2] Department of Microbiology & Immunology, Dalhousie University, Halifax NS Canada

Supplementary Figure S1. qPCR validation of genes identified by microarray. Gene-specific qPCR primers were used to validate genes regulated by ALDH1A3 in (a) MDA-MB-231 and MDA-MB-468; (b) MDA-MB-231 alone; and (c) MDA-MB-468 alone. Gene-specific primers were also used to validate the genes regulated by atRA in (d) MDA-MB-231 and MDA-MB-468; (e) MDA-MB-231 alone; and (f) MDA-MB-468 alone. Statistical significance was determined via paired student's t-test (n=4; * p<0.05; ** p<0.01; *** p<0.001).

Supplementary Figure S2. Hierarchical clustering of all genes regulated by atRA or ALDH1A3 in one or both cell lines. Z-scores for each gene listed in the heatmap were taken and clustered by gene and sample using the heatmap.2 function in the R environment.

Supplementary Figure S3. DAC does not alter the atRA-inducibility of GPRC5B and IQGAP2. qPCR was performed for GPRC5B and IQGAP2 in MDA-MB-231 and MDA-MB-468 cells following treatment with combinations of atRA, decitabine and trichostatin A. Statistical significance was determined via a 2-way ANOVA (n=4; * p<0.05; ** p<0.01; *** p<0.001).

Supplementary Figure S4. Changes in expression following DAC or atRA treatment are not accompanied by corresponding changes in DNA methylation. (a) HOXB2 and CD22 expression was measured by qPCR in MDA-MB-231 and MDA-MB-468 cells following treatment with combinations of atRA, decitabine and trichostatin A. Statistical significance was determined via a 2-way ANOVA (n=4; * p<0.05; ** p<0.01; *** p<0.001). (b) Methylation of HOXB2 and CD22 was compared following DAC treatment using the HumanMethylation450 array (n=3). (c) Expression of TINAGL1 and PRDM1 was measured by qPCR in MDA-MB-231 and MDA-MB-468 cells following treatment with combinations of atRA, decitabine and trichostatin A. Statistical significance was determined via a 2-way ANOVA (n=4; * p<0.05; ** p<0.01; *** p<0.001).

Supplementary Figure S5. Transcription factors associated with atRA-downregulated genes are largely unrelated between MDA-MB-231 and MDA-MB-468 cells. Transcription factors (TFs) identified by PASTAA analysis were compared between cell lines and the lack of overlap is demonstrated in a Venn diagram.

Supplementary Figure S6. Hierarchical clustering of transcription factors associated with atRA-upregulated genes in MDA-MB-231 or MDA-MB-468 cells. Z-scores for each transcription factor identified by PASTAA analysis (as in Figure 4) were taken and clustered by gene using the heatmap.2 function in the R environment.

Supplementary Figure S7. IRF1 knockdown affects the expression of IRF1-target genes RARRES3 and GBP4 but not TNFSF10. (a) RARRES3 expression is compared between MDA-MB-231 and MDA-MB-468 cells by qPCR. Knockdown of IRF1 (as in Figure 5a) has a significant effect on RARRES3 expression. (b) GBP4 expression is compared between MDA-MB-231 and MDA-MB-468 cells by qPCR. Knockdown of IRF1 (as in Figure 5a) has a significant effect on GBP4 expression. (c) TNFSF10 expression is compared between MDA-MB-231 and MDA-MB-468 cells by qPCR. Knockdown of IRF1 (as in Figure 5a) has a significant effect on TNFSF10 expression. Statistical significance was determined by paired student's t-test (left panel) or by two-way ANOVA with repeated measures (centre and right panels). For all comparisons, n=4; * p<0.05; ** p<0.01; *** p<0.001.

Supplementary Table S1. Primers used for qPCR

	Forward (5' -> 3')	Reverse (5' -> 3')
GAPDH[8]	GGAGTCAACGGATTTGGTCGTA	TTCTCCATGGTGGTGAAGAC
B2M[78]	AGGCTATCCAGCGTACTCCA	CGGATGGATGAAACCCAGACA
GDF15	TCCAGATTCCGAGAGTTGCG	CGAGGTCGGTGTTCGAATCT
CDH5	CTTCACCCAGACCAAGTACACA	TCAACAAACAGAGAGCCCACA
SCEL	CCAAAGTCTCGACAGCCTCA	CTGCTTTTGTTTGCTGAAGGGA
IRF1	TCGGATGCGCATGAGACC	CATGCTTCCATGGGATCTGGA
STAT1	TGCCAGCCTGGTTTGGTAAT	GTACCAAAGGATGGAGGCCC
CTSS	ACATGGGTTCTTGTGGTGCT	AGTTGAGCAATCCACCAGGTT
GBP4	TCCTTGACATGGCTAGCAACA	GCCAAGATATTTTGTCCCTACTCC
RARRES3	GGCTGTTGCTATCGGGTCAA	GACCAACCATCTCCTTCGCA
TNFSF10	TGCGTGCTGATCGTGATCTT	CTGCTTCAGCTCGTTGGTAAAG
DHRS3	TCTGTGATGTGGGCAACCG	ATGGTGATGTCACCCACCTTC
NRP2	CACCAGAACTGCGAGTGGAT	TGCAGTCGTGCTTCTCGATT
PTGES	CTTTTGTCGCCTGGATGCAC	GTAGGTCACGGAGCGGATG
RARB	GGTTTCACTGGCTTGACCAT	GGCAAAGGTGAACACAAGGT
AMPH	ATCGTATTGAGTGGCTGCCC	CACCTGGGCAGGGGAATAAG
SSX1	GAAGCCAGCAGAGGACGAAA	CAGAAATATTTGCTTTTCCTGGGGG
ESM1	CGGTTCTGGGGCATAGGAAA	ACACAAACCACCAGTGGGTAA
ARL14EPL	ACCCATGCCCGAAGTGTAAC	CCTGACTCAGTGACGATGGC
CST2	AGGAGGACAGGATAATCGAGGG	AGTGGCCTTGTTATACTCGCTG
LTBP3	AGATCTCAGCAGAAGTGCAGG	TTCGAGCTCTCAATGCGGT
TINAGL1	CCTTTTCCCCCGATCCAAGG	GGTTCTTGGTCACACTGCCA
CRISP2	GGTTGCCCTGATGACTGTGA	TGTTCACAGCCAGCTGTATTCT
CRISP3	AAATACTTCATCCTGCTCTGGAAAC	GCAGTAAAAGCGGGATCCTTATC
S100A7	CCAAGCCTGCTGACGATGA	GACATCGGCGAGGTAATTTGTG
COLCA2	TGTCGGAAAAACCGAAGGTGTA	CCTGGTGTGCCCGTCTTT
CD22	TGGGAGAAAAATGGCAGGCT	GGACGCTGTCTGTCCTATGG
FOXA1	CATGAAACCAGCGACTGGAAC	TCATGTTGCTGACCGGGAC
NFKBIZ	GTCCGCCTGTTGATGAGGAA	GGAACCAAATGCACTGGCTG
SRPX2	ACCGGGGCTTTCGATTGATT	TGAATGGTAGTGCGTGGCAT
TMPRSS4	TGAAGCTGCAGTTCCCACTC	TTCTGCTTCGTAAAGCCCCAT
LINC00857	GAGGCCCTAATCCTCAAGGC	TCTTTTCCTTCACACCGCGT

Supplementary Table S2. Genes with identified RARE sequences.

Gene	RARE sequence
Associated with atRA-upregulated genes in MDA-MB-231	
ELF3	AGGTCAGAGGGAGGTCA
GBP4	TCAACTTGGAATGAACT
DHRS3	GGTTCAGCCACAGGTCA
	GGGTCATGGAGAGGTCA
	AGGTCAGGGGAAGGACT
LTBP3	GTGTCATTGGGAGGTCA
NRIP1	AGGGCACCTGCAGTTCA
	AGTTCAACAGGAGGTAA
	AGGTCATTTAGAGGACA
	AGGTCACACAAAGGAGA
NRP2	AGGTCACTAAGGGGTCA
	AGTTCATTAAAATGTCA
	GGATCACAAAGAGGTGA
RARB	GGGTCATTTGAAGGTTA
	GGTTCACCGAAAGTTCA
	GGGTCACGGGCAGGTTA
	AGTTCAACTAAAGTACA
TINAGL1	GGTTTGCAGGGAGGTCA
Associated with atRA-upregulated genes in MDA-MB-468	
ADH1C	GGGTCATTCAGAGTTCA
CYP26A1	GGGTCACAGGCGGGTCA
CYP27A1	TGAACTTCTCTTCACCT
FOXA1	TGACCTCCTCATGAACC
	AGGTCAGGGGGGAGGGGA
SRPX2	TGACCTAAAGGTGAACT
STRA6	AGGTGAACCCAAGTTCA
TRIM31	AGGTCACAGCCAGTTCA
ARHGEF6	GGGTCAGGGGAAGGGGA
CLUL1	AGGTCACGCAAAGTTTA
CYP26A1	GGTTCACTAAGGGGTCA
	AGTTCACTCGGATGTCA
	GGGTCACAGGCGGGTCA
DHRS3	GGTTCAGCCACAGGTCA
	GGGTCATGGAGAGGTCA
	AGGTCAGGGGAAGGACT
EPB41L4A	AGGTCAGCATCAGGGCA
	GGTTCATGAGGAGGTCA
HOXB2	GGTTCAAGAAGAGTTCA
IFFO2	GGGTGTGGGGGAGGTCA
LRG1	TGGTCAGCTGGAGGTCA
	AGGGCAGGGGAGGGTCA
NAV2	TGGTCACTCACAGGTCA
	GGGGCAGGGACAGGTCA
NRP2	AGGTCACTAAGGGGTCA
	AGTTCATTAAAATGTCA
	GGATCACAAAGAGGTGA
PELI2	GGGTCACACACAGTTCA

Gene	RARE sequence
Associated with ALDH1A3-upregulated genes in MDA-MB-231	
CCL2	TGACCCCTCCTTCACCC
PTGFRN	TGAACCATTGATGACCC
DHRS3	GGTTCAGCCACAGGTCA
	GGGTCATGGAGAGGTCA
	AGGTCAGGGGAAGGACT
LTBP3	GTGTCATTGGGAGGTCA
NRIP1	AGGGCACCTGCAGTTCA
	AGTTCAACAGGAGGTAA
	AGGTCATTTAGAGGACA
	AGGTCACACAAAGGAGA
NRP2	AGGTCACTAAGGGGTCA
	AGTTCATTAAAATGTCA
	GGATCACAAAGAGGTGA
RARB	GGGTCATTTGAAGGTTA
	GGTTCACCGAAAGTTCA
	GGGTCACGGGCAGGTTA
Associated with ALDH1A3-upregulated genes in MDA-MB-468	
ALDH2	AGGTCAAGCTGAGTTGA
CEBPA	TGAACCAGAATTCACCC
CYP27A1	TGAACTTCTCTTCACCT
GSTA2	TCACCCTCGCCTGAACC
S100A7	GGGTGAAGTTGGGGTGA
S100A7A	TCACCCCAACTTCACCC
ARHGEF6	GGGTCAGGGGAAGGGGA
CACNA1D	GGGTTAGTGAGAGGTCA
	AGGACACGGAGAGGTCA
DHRS3	GGTTCAGCCACAGGTCA
	GGGTCATGGAGAGGTCA
	AGGTCAGGGGAAGGACT
EPB41L4A	AGGTCAGCATCAGGGCA
NFKBIZ	GGGTCATGGTGAAGTGA
PELI2	GGGTCACACACAGTTCA

Supplementary Table S3. Putative methylated atRA-inducible genes.

Cluster	Gene Symbol	RA-upregulated in?	RARE?
i	ST3GAL1	MDA-MB-468	
	RASSF6	MDA-MB-468	DR2[29]
	TP53INP1	MDA-MB-468	
ii	SLC46A3	MDA-MB-468	
	AGR2	MDA-MB-468	
	GPRC5B	MDA-MB-468	
	LOC105378744	MDA-MB-468	
iii	HLA-B	MDA-MB-231	
	LOC105373682	MDA-MB-468 / MDA-MB-231	
	TINAGL1	MDA-MB-231	DR5 (oPOSSUM)
	IQGAP2	MDA-MB-468	
	HOXB2	MDA-MB-468	DR5 (oPOSSUM)
	TNFSF15	MDA-MB-468	
	PRDM1	MDA-MB-468	
	SRPX2	MDA-MB-468	DR5[25]
	LOC105375401	MDA-MB-468	
	TFPI2	MDA-MB-468	
iv	LOC105376382	MDA-MB-468	
	SAMD9	MDA-MB-468	
	LINC00857	MDA-MB-231	

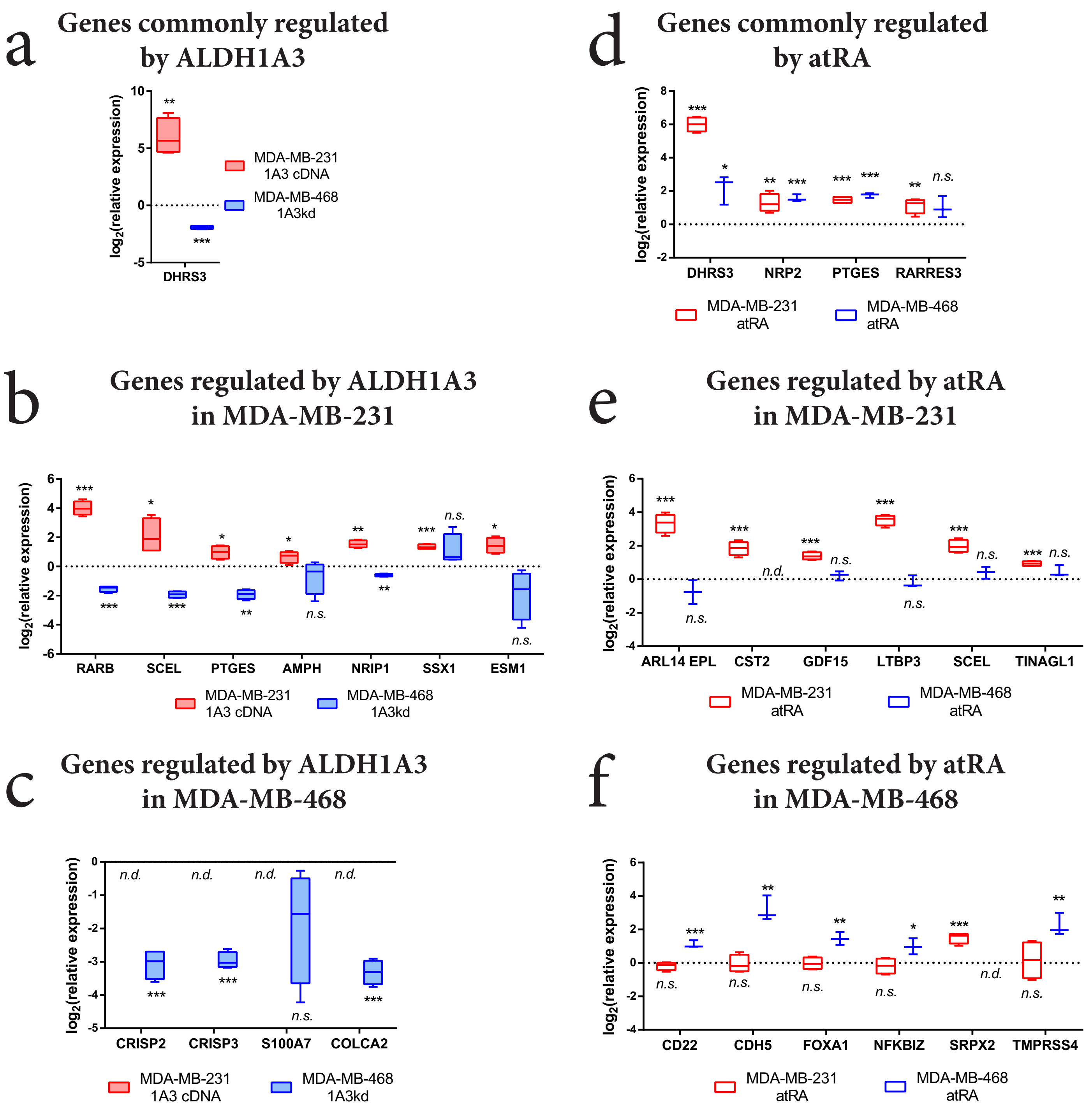

Supplementary Figure S1

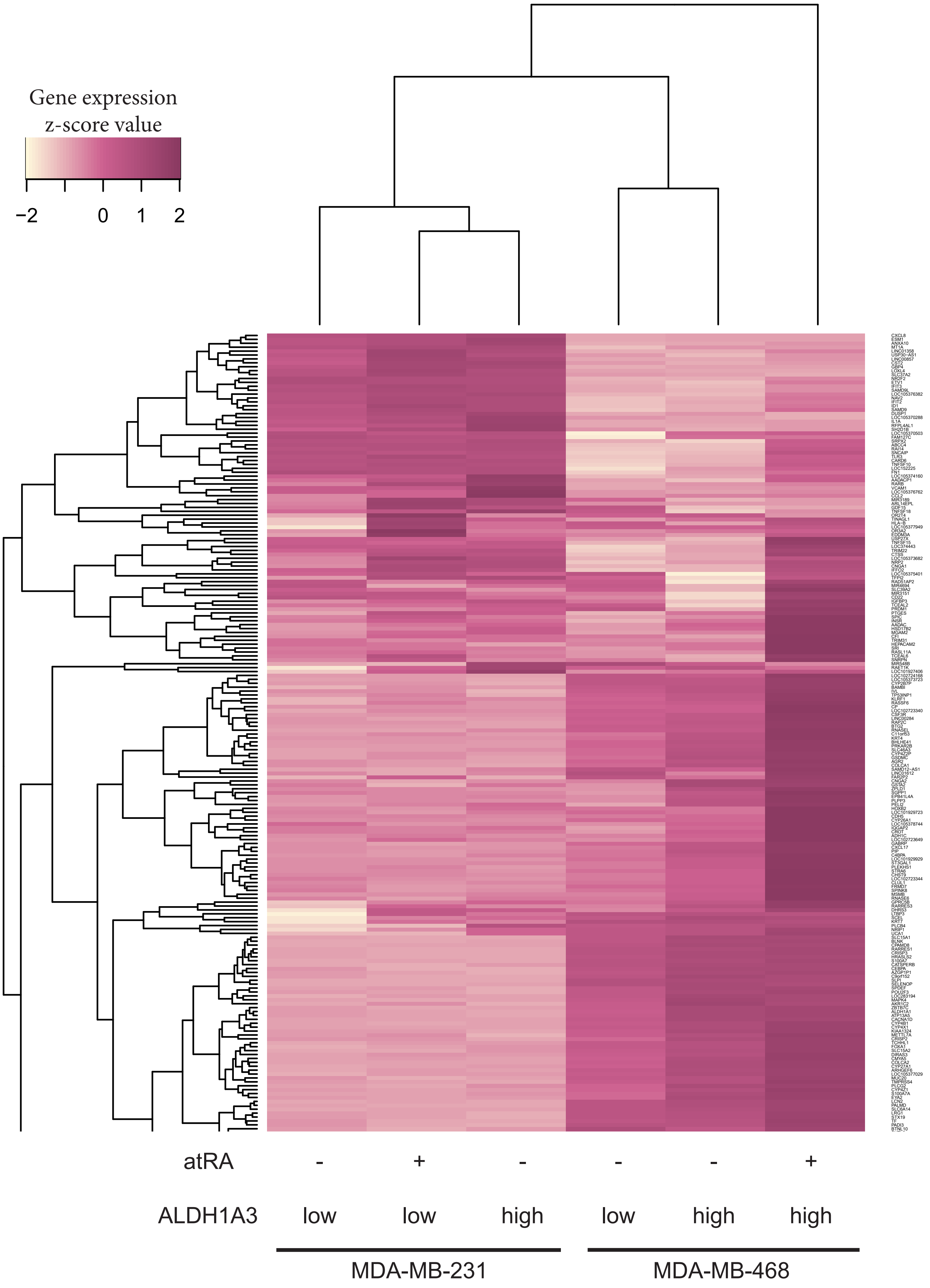

Supplementary Figure S2

Expression of atRA-inducible genes after DAC and/or TSA treatment

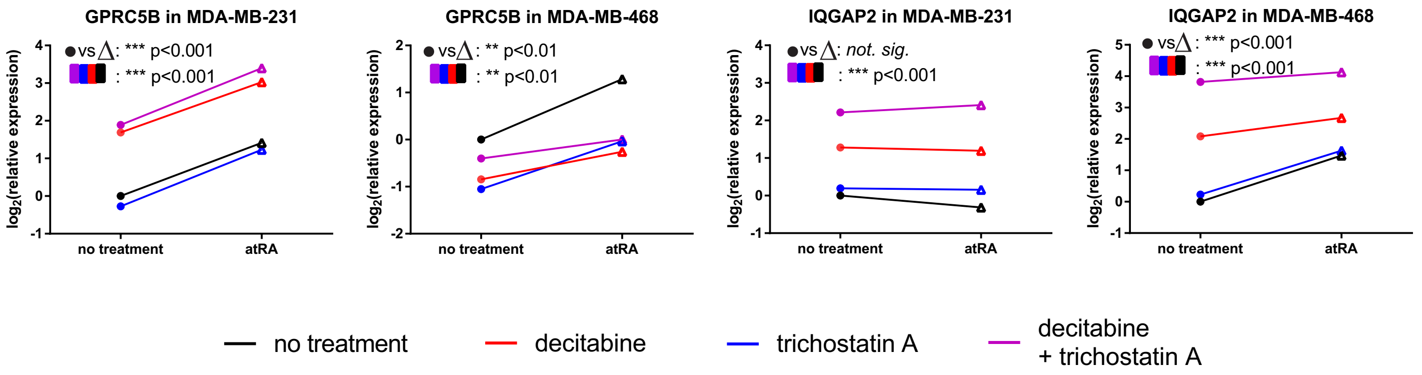

Supplementary Figure S3

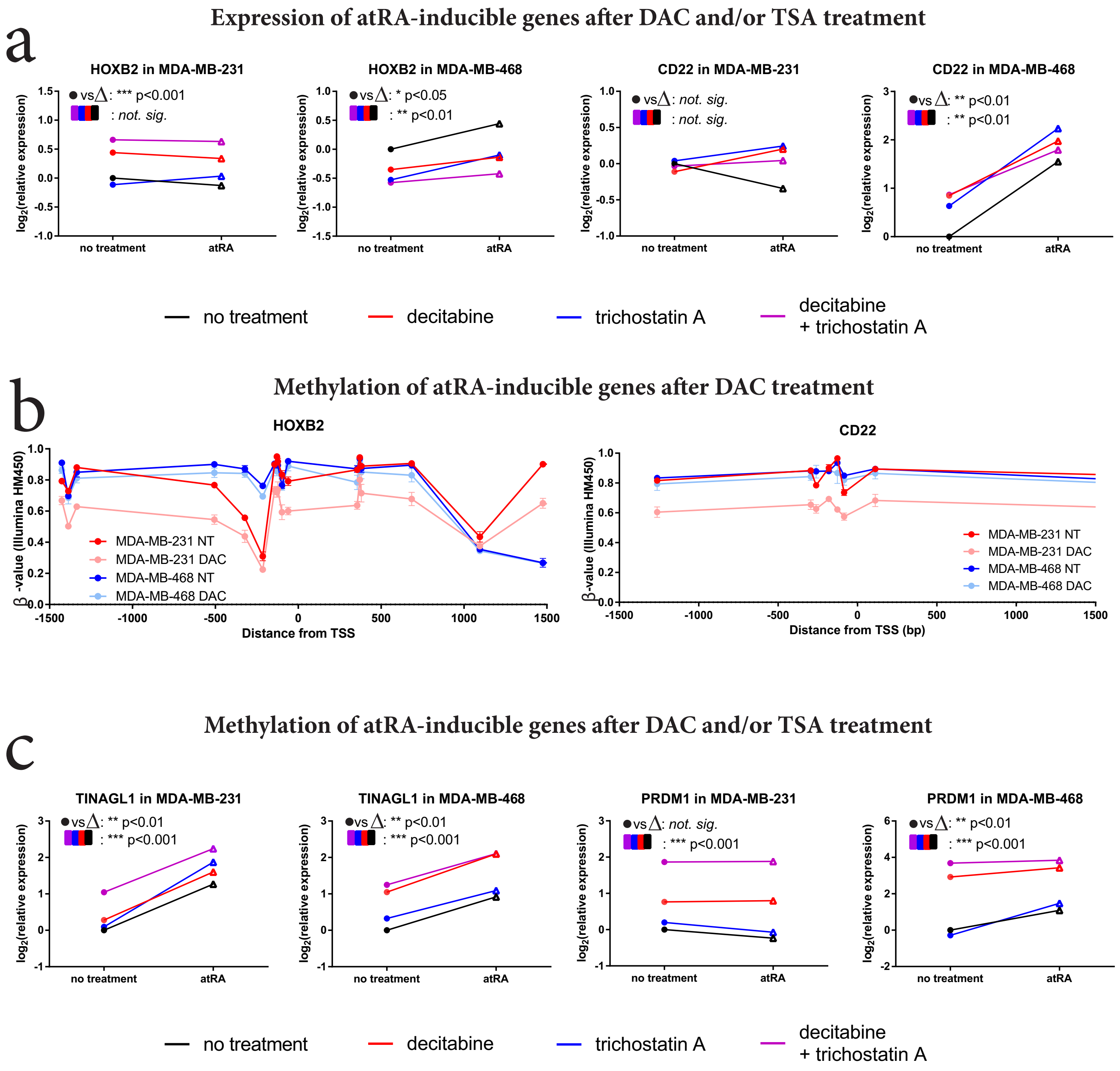

Supplementary Figure S4

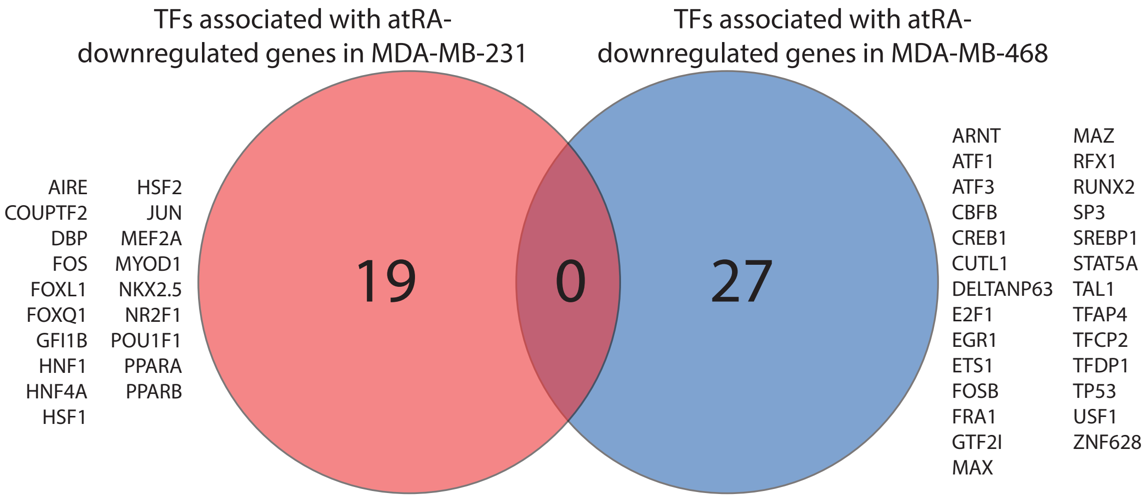

Supplementary Figure S5

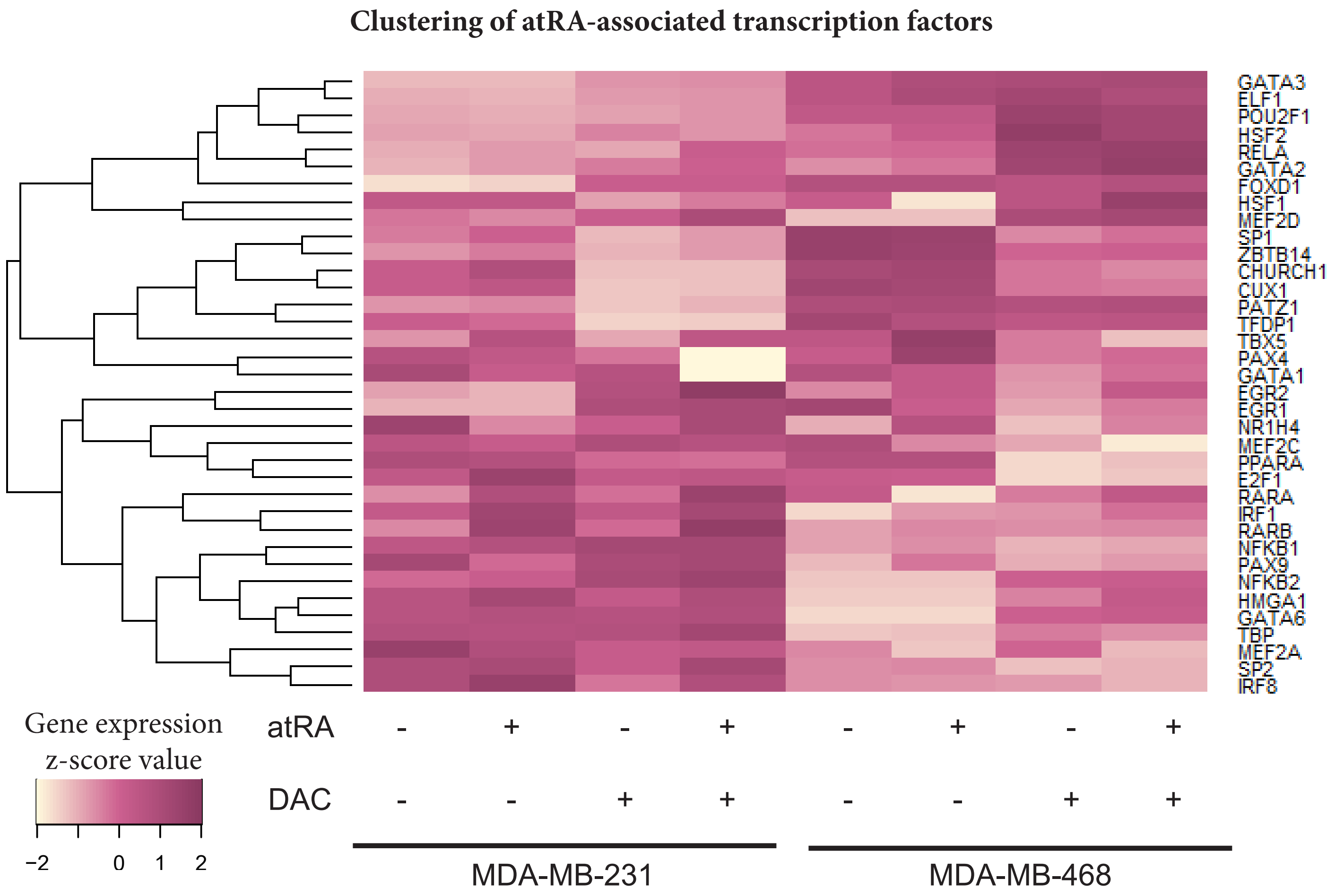

Supplementary Figure S6

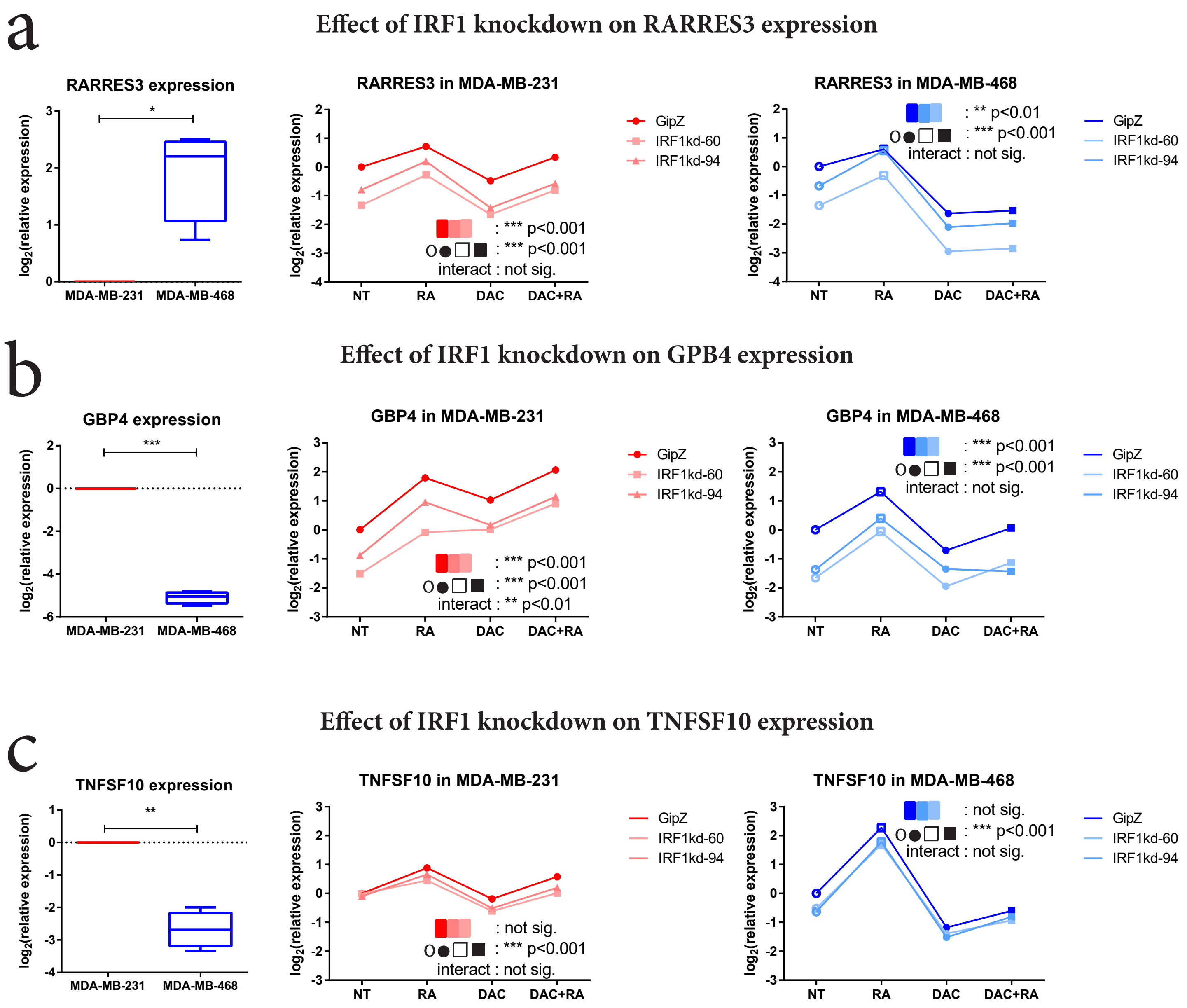

Supplementary Figure S7
